# The Role of Orbital Atherectomy for Complex Coronary Calcium Modification: Has It Been Eclipsed?

**DOI:** 10.3390/jpm15090414

**Published:** 2025-09-02

**Authors:** Natasha Khullar, Trisha Singh, Peter O’Kane, Jonathan Hinton

**Affiliations:** Dorset Heart Centre, Royal Bournemouth Hospital, Castle Lane East, Bournemouth BH7 7DW, UKtrisha.singh@uhd.nhs.uk (T.S.); peter.o'kane@uhd.nhs.uk (P.O.)

**Keywords:** percutaneous coronary intervention, coronary calcification, orbital atherectomy

## Abstract

Severe coronary artery calcification (CAC) is a frequent finding in patients undergoing percutaneous coronary intervention (PCI) and represents a significant procedural challenge. CAC is commonly associated with ageing and comorbidities such as diabetes, hypertension, and chronic kidney disease, and contributes to vessel rigidity, impaired device delivery, and suboptimal stent expansion. These factors increase the risk of angiographic complications, as well as major adverse cardiac events compared with non-calcified lesions, negatively impacting both immediate and long-term clinical outcomes. In cases of severe calcification, traditional balloon angioplasty is often inadequate, necessitating the use of dedicated calcium modification techniques. Devices such as rotational atherectomy (RA), orbital atherectomy (OA), excimer laser coronary atherectomy (ELCA), and intravascular lithotripsy (IVL) have been developed to address these challenges. Among these, orbital atherectomy offers a potential unique dual mechanism of action and has shown promise in enhancing lesion preparation and facilitating optimal stent deployment. This review provides an overview of the role of orbital atherectomy in the management of calcified coronary lesions, evaluates current evidence on its safety and efficacy, and discusses how it may be positioned in the future, underscoring the need for a personalised, lesion-specific approach to optimise PCI outcomes.

## 1. Introduction: The Problems of Calcium in PCI

Coronary artery calcification (CAC) is characterised by the accumulation of calcium phosphate deposits and is frequently identified in both invasive coronary angiography and computerised tomography [1]. Traditional risk factors include advanced age and smoking, while the rising prevalence of comorbidities, such as diabetes mellitus, systemic hypertension and chronic kidney disease, have contributed to the increased frequency and severity of CAC [2,3]. These conditions impair endothelial integrity, triggering inflammation through leukocytes and vascular smooth muscle cells, culminating in ectopic calcification within the intimal and medial layers of the arterial wall [4,5].

CAC presents significant challenges to percutaneous coronary intervention (PCI) by both impairing device delivery and increasing arterial wall rigidity, which can potentially result in suboptimal stent deployment and expansion [6,7,8]. This increases the risk of PCI-associated complications, such as, in-stent restenosis requiring target lesion revascularisation (TLR), stent thrombosis, and stent malapposition, all of which are more frequent in complex, long or bifurcation calcified lesions [6,7,9,10,11,12,13,14,15]. These lesions are also prone to complications such as vessel dissection, slow/no-reflow, device embolisation, and vessel perforation—especially when high-pressure post-dilatation is employed to address stent underexpansion [16,17]. The presence of severe calcification remains a key predictor of adverse outcomes, including reduced procedural success rates and higher incidences of major adverse cardiovascular events (MACE) [10,18,19,20,21]. The MACE-Trial (Multi-centre Prospective Study to Evaluate Outcomes of Moderate to Severely Calcified Coronary Lesions) demonstrated that patients with severe CAC had lower procedural success (86.8%) compared to those with moderate (95.0%) or none/mild calcification (97.7%) [20]. At one year, MACE rates were markedly higher in the severe group (24.4%) versus moderate (8.7%) and none/mild (4.7%) [20]. Additionally, a pooled analysis of seven contemporary stent trials revealed significantly higher all-cause mortality at three years in patients with severe CAC (10.8% vs. 4.4%; *p* < 0.001), along with a higher incidence of the combined endpoint of death/MI (22.9% vs. 10.9%; *p* < 0.001) [21].

Conventional balloon angioplasty techniques often prove ineffective in the presence of severe calcification, necessitating the use of advanced calcium modification tools to improve procedural outcomes [22]. These calcium modification techniques, including rotational atherectomy (RA), orbital atherectomy (OA), excimer laser coronary atherectomy (ELCA) and intravascular lithotripsy (IVL), have shown promise in enhancing PCI outcomes with low periprocedural complication rates [22,23,24,25,26,27,28]. RA and ELCA have been available for several years, while OA, though well established in the United States over the last three decades, has only recently gained approval for use in Europe in 2021. Despite the options available, atherectomy devices are frequently underutilised. Registry data indicates that only 2.0% of all PCIs performed in the United Kingdom involved coronary atherectomy in 2019, even though it has been suggested that the prevalence of CAC in the PCI patient population is around 20% [29,30]. Whilst accepting that many of these cases of CAC will not need coronary atherectomy, there is still marked discordance between the prevalence of CAC and atherectomy usage, thus highlighting the importance of taking a patient- and lesion-focused approach to device selection. This variability in atherectomy usage may be due to factors such as operator experience, perceived procedural inefficiency/length of procedure, device availability and increased cost. Additionally, the emergence and rapid evolution of IVL has greatly influenced current practice, contributing to a decline in atherectomy use in favour of IVL [31,32,33]. A comparison of the available calcium modification devices is shown in Table 1. Beyond these specialized calcium modification devices there are balloon-based devices that can also play a role in the modification of calcification including cutting, scoring and OPN balloons. Whilst these devices have a role, if a balloon-based approach is feasible then IVL is likely to be preferable due to its efficacy and excellent safety profile, resulting from the low-pressure nature of IVL inflations.

The purpose of this review is to (a) provide an update on the current role of orbital atherectomy in CAC, (b) discuss the latest trials/studies regarding its safety and efficacy, and (c) highlight the need for further research to provide a personalised approach, based on specific patient and lesion characteristics, for the management of CAC.

## 2. Orbital Atherectomy: Mechanism of Action

**Figure 1:**
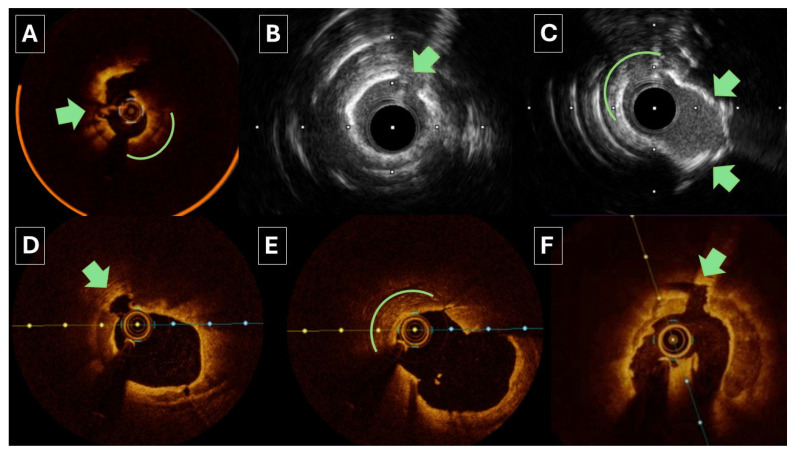
Intravascular imaging findings immediately following orbital atherectomy. Green arrows highlight fracturing and green curves highlight smoothing of calcification as a result of orbital erosions. Panel (**A**) demonstrates fracturing and somewhat eccentric erosion of calcium in LAD. Panel (**B**,**C**) demonstrate fracturing following orbital atherectomy with panel (**C**) demonstrates some eccentric erosion due to wire bias. Panel (**D**,**E**) are from a proximal/ostial circumflex lesion with panel (**D**) showing deep fracturing and panel (**E**) showing significant eccentric erosion as a result of marked wire bias. Panel (**F**) shows deep fracturing following orbital in an LAD.

The Diamondback 360° Coronary Orbital Atherectomy System (OAS) provides an additional treatment option for managing severely calcified coronary lesions, particularly in cases where conventional balloon angioplasty may be undeliverable, fail to fully expand or be insufficient for adequate lesion preparation [16,22,34,35,36]. When used as a bail-out strategy in balloon-uncrossable or undilatable lesions, OA offers advantages over RA, especially in the context of eccentric calcification, suboptimal wire bias, or when calcium is located adjacent to a recently deployed stent or at a bifurcation [22,34,37,38,39]. In this setting, OA facilitates circumferential and controlled plaque modification, potentially mitigating the risk of wire bias-related vessel injury [37,38,39]. The OAS employs a centrifugal, differential sanding mechanism to modify calcified plaque while preserving healthy arterial tissue [40,41]. Central to its function is a diamond-coated 1.25 mm crown mounted on a shaft driven by an electrically powered motor, which rotates at two different, operator selected speeds (80,000 and 120,000 revolutions per minute). The centrifugal force generated during activation enables the crown to orbit eccentrically within the vessel, selectively ablating rigid, calcified material while sparing the more elastic, non-calcified components of the vessel wall. This targeted ablation reduces the risk of vessel injury and thermal damage, thus improving the safety profile of the procedure [36,42,43]. In addition to the calcium ablation from the OAS, the orbital motion of the crown induces micro-fractures within the calcified plaque, increasing compliance and facilitating subsequent balloon angioplasty and stent deployment [35]. This potential dual mechanism of action, both ablative and fracturing of CAC, offers an exciting strategy for potentially improving CAC lesion preparation (Figure 1).

Pre-procedural assessment with intracoronary imaging—typically intravascular ultrasound (IVUS) or optical coherence tomography (OCT)—is recommended to characterise lesion morphology and quantify calcium burden, assuming that the intracoronary imaging device is deliverable, which is frequently not possible in severe CAC [36,44]. On OCT, lesions exhibiting calcium deposits with a maximal arc > 180°, thickness > 0.5 mm, and length > 5 mm are associated with an increased risk of stent underexpansion [44]. Once the lesion has been appropriately assessed, the OAS is advanced over the viper guidewire to the site of calcification. Upon activation, the crown orbits within the vessel, with atherectomy speed and time adjusted by the operator to optimise plaque modification [42,43].

**Figure 2:**
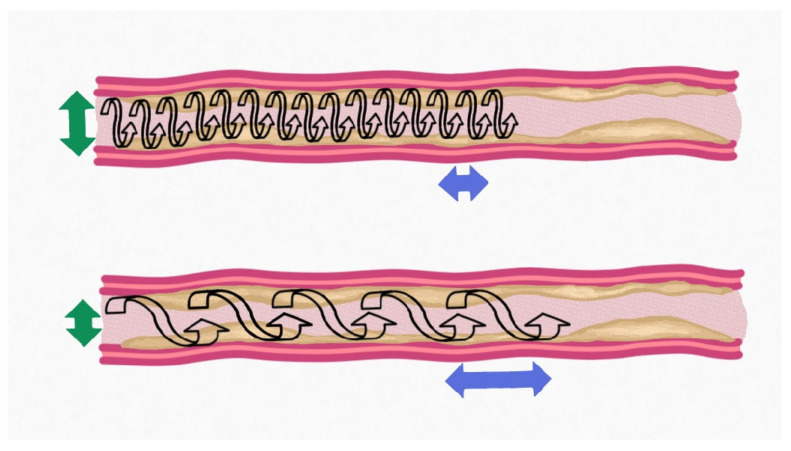
Impact of slow operator motion of orbital atherectomy crown. **Top panel**—slow motion, allowing coverage of entire length of vessel and increasing radial orbit of device. **Bottom panel**—fast motion, resulting in missing longitudinal areas of the vessel and incomplete opening of orbit of device resulting in suboptimal preparation.

The OAS offers distinct advantages in the management of complex coronary lesions, including those located in proximal segments or bifurcation sites, where alternative atherectomy systems, such as rotational or directional devices, may be less effective [22,34,36]. It is most commonly used in the left anterior descending artery (LAD), but also in the right coronary artery (RCA), left circumflex artery (LCx), and at ostial or bifurcation sites [16]. LAD lesions are the most frequent targets due to both the high prevalence of calcification and the clinical importance of optimal stent expansion in this territory. OA is also effective for technically challenging ostial lesions and bifurcations, with real-world data supporting its safety and high procedural success [39,45,46]. A key feature of the OAS is the ability of the operator to utilise the device in both antegrade and retrograde directions, providing several procedural benefits: (a) an initial retrograde approach minimises the risk of an abrupt and uncontrollable forward jump of the device, (b) alternating between the antegrade and retrograde passes can alter wire bias, potentially improving lesion preparation, particularly at angulated bifurcations such as the ostium of the circumflex artery, and (c) the bidirectional approach may reduce the risk of entrapment [35]. Despite the orbital nature of the device, significant wire bias can lead to focused asymmetrical erosion of calcification (Figure 1). Luminal gain can be progressively increased by extending the duration of crown-to-lesion contact, increasing the number of device passes, or by adjusting the rotational speed [36]. The Society for Cardiovascular Angiography & Interventions recommends starting orbital atherectomy at 80,000 rpm in all vessels [47]. Higher speed (120,000 rpm) can be utilised following this in larger vessels (<3.5 mm) and can help improve the degree of modification. Given that the crown orbits the vessel, it is important that the operator only move the device slowly for two reasons; firstly, a slower operator motion allows the device to fully open up the diameter of its orbit, and second, a slower motion means that the device engages with the entire vessel rather than skipping areas (Figure 2). This increases the duration of crown-to-lesion contact per orbit, thereby increasing the extent of calcium ablation [47]. Continuous coronary flow during orbital atherectomy aids in the washout of ablated debris and provides thermal regulation of the crown, thereby minimising the risk of distal embolisation and heat-related vascular injury [36,40,42,48]. The average particle size created by the OAS is small (2.04 µm) which helps to reduce the risk of slow flow [49]. Once adequate plaque modification has been achieved, PCI is completed with balloon angioplasty as needed, followed by implantation of destination therapy [36,48].

### 2.1. Orbital Atherectomy: Clinical Studies

Three major clinical trials (ORBIT I, ORBIT II, ECLIPSE), a retrospective multicentre registry (Lee et al.), a prospective registry (LOAR), a multicentre prospective study on the Micro Crown OAS (COAST), a single-centre retrospective study (Helal et al.), and a comparative randomised trial on RA vs. OA (DIRO) have been published on the OAS (Table 2). Multiple subanalyses from the ORBIT II trial have also been published, exploring post-orbital atherectomy outcomes across various clinical subgroups, including stratifications by gender, diabetes, advanced age, left main disease, renal dysfunction, and left ventricular systolic dysfunction [36,50,51,52,53,54,55,56,57,58].

Atherectomy is associated with potential complications, including dissection, perforation, distal embolisation, vessel closure, slow flow or no-reflow, spasm, and thrombus formation [36,41,42,43,47,59,60,61,62,63,64]. The safety profile of the OAS has been explored in the studies presented (Table 3). Among these outcomes, dissection and perforation are the most prevalent adverse events associated with OA, with frequency and severity varying based on lesion characteristics, vessel size, and the specific procedural technique employed [41,47,65].

#### 2.1.1. ORBIT I Trial

The orbital atherectomy device was first evaluated in the coronary space in ORBIT I, a prospective, non-randomised study designed to evaluate the safety and feasibility of the OAS for the treatment of de novo calcified coronary lesions. A total of 50 patients were enrolled across two centres in India. The primary aim was to assess the capability of the OAS to modify heavily calcified plaque and facilitate stent delivery while minimising procedural complications. The mean age of the study population was 57.4 years, with a predominance of male patients (90%); the average lesion length was 13.4 mm, and the mean number of OA devices used per procedure was 1.3. Device success was reported in 98% of cases, while procedural success, defined as successful stent delivery with residual stenosis < 50% without in-hospital MACE, was achieved in 94% of patients [41].

The cumulative MACE rate was 4% during index hospitalisation, 6% at 30 days, and 8% at six-month follow-up. At three-year follow up, the frequency of TLR was low (3.3%) and the overall MACE rate was 18.2% [26]. Angiographic complications included six coronary dissections and one vessel perforation (Table 3). Despite these events, the study concluded that OA may serve as an effective strategy for modifying lesion compliance in severely calcified vessels, thereby facilitating optimal stent delivery and expansion. However, the investigators acknowledged the limitations inherent to a small, non-randomised cohort and underscored the need for larger, controlled trials to more definitively establish the safety and efficacy of orbital atherectomy in this high-risk patient subset. Furthermore, although the 1.25 mm crown is the only size currently approved for coronary use, it was utilised in just 18.4% of patients in the ORBIT I trial, with the remaining majority (>80%) of patients in the trial treated with crown sizes that are no longer available for use in coronary interventions [41]. This limits the direct applicability of the ORBIT I trial’s findings to current clinical practice.

#### 2.1.2. ORBIT II Trial

ORBIT II (Evaluate the Safety and Efficacy of OAS in Treating Severely Calcified Coronary Lesions) was a larger prospective, multicentre, non-blinded study which enrolled 443 patients across 49 sites in the United States, evaluating the safety and efficacy of the OAS in patients with de novo, severely calcified coronary lesions. The primary safety endpoint was defined as freedom from MACE at 30 days, comprising cardiac death, MI, and target vessel revascularisation (TVR). The primary efficacy endpoint was procedural success, defined as successful stent delivery with residual stenosis < 50% in the absence of in-hospital MACE [43].

The study demonstrated that the OAS was effective in modifying calcified lesions, facilitating stent delivery in 97.7% of cases, with a significant increase in mean minimal lumen diameter from 0.5 mm pre-procedure to 2.9 mm post-procedure on quantitative coronary angiography (QCA) [43]. The primary safety endpoint was met, with 89.6% of patients free from MACE at 30 days, and the primary efficacy endpoint was achieved in 88.9% of patients [43]. At one-year follow-up, the cumulative MACE rate was 16.4%, including cardiac death in 3.0%, MI in 9.7%, and TVR in 5.9% of patients [42]. The TLR rate was 4.7%, and stent thrombosis was observed in only 0.2% of the cohort at one year [42]. Multivariate analysis identified baseline diameter stenosis and the use of bare-metal stents as independent predictors of one-year MACE and TVR [42]. At three-year follow-up, the rate of TVR was 7.8%, which is relatively low considering the complexity of the cases undertaken with the OAS [25].

Although the single arm design was a limitation, ORBIT II demonstrated that OA is both a safe and effective strategy for the preparation of severely calcified coronary lesions. The trial supports its use in enhancing stent delivery and improving short- and mid-term clinical outcomes, reinforcing the role of OA as an important adjunctive modality in the management of complex calcified coronary artery disease.

#### 2.1.3. OAS Real-World Multicentre Registry

A retrospective multicentre study by Lee et al. evaluated the safety and efficacy of orbital atherectomy prior to stent placement in a real-world cohort of patients with severe CAC [59]. This study included 458 consecutive patients treated with OA at three centres in the United States from October 2013 to December 2015. Patients were enrolled based on severe CAC determined by angiography [59].

The primary endpoint of 30-day major adverse cardiac and cerebrovascular events was 1.7%. The study reported low rates of 30-day all-cause mortality (1.3%), MI (1.1%), TVR (0%), stroke (0.2%), and stent thrombosis (0.9%). Angiographic complications were also minimal, with perforation occurring in 0.7% of cases, dissection in 0.9%, and no-reflow in 0.7% (Table 3). Emergency coronary artery bypass graft surgery was required in only 0.2% of patients [59].

In this large real-world study of patients undergoing orbital atherectomy, the OAS was found to be a safe and effective tool for modifying severe CAC, facilitating stent delivery and optimal expansion in complex coronary anatomy. The study demonstrated the low incidence of acute and short-term adverse clinical events in a high-risk population, including patients who were not surgical candidates and who would have been excluded from the ORBIT II trial. Considering the nonrandomised, retrospective design, the authors concluded that a randomised clinical trial is needed to identify the ideal treatment strategy for patients with severe CAC [59].

#### 2.1.4. COAST Study

The Coronary Orbital Atherectomy System Study (COAST) evaluated the safety and efficacy of the Micro Crown OAS for the preparation of severely calcified coronary lesions before stent implantation [60]. This OA device incorporates a tapered micro crown with a diamond-coated tip in addition to the classic crown (1.25 mm), designed to work at lower rotational speeds (50,000 or 80,000 rpm). The prospective, multicentre, single-arm study enrolled 100 patients with severely calcified de novo coronary lesions at 17 sites in the United States and Japan. The study’s primary effectiveness endpoint was procedural success, defined as stent delivery with residual stenosis < 50% without in-hospital MACE, and the primary safety endpoint was freedom from MACE at 30 days [60].

This registry found that the Micro Crown OAS facilitated stent delivery and residual stenosis < 50% to be achieved in 99.0% of cases. Procedural success was achieved in 85.0% of subjects, and freedom from MACE at 30 days was 85.0%. At one year, freedom from MACE was 77.8% [60]. These findings suggest that the Micro Crown OAS is a safe and effective tool for the preparation of severely calcified coronary lesions, with the study’s outcomes comparable to those observed for the Classic Crown OAS in the ORBIT II trial [41,42,43]. Nonetheless, the Micro Crown OAS is not readily available in Europe, representing a limitation on broader applicability.

#### 2.1.5. LOAR Registry

In contrast to the more controlled, prospective designs of the COAST and ORBIT II trials, the Lower Silesian Orbital Atherectomy Registry (LOAR) assessed the safety and efficacy of OA in an all-comers population presenting with severely calcified coronary lesions [42,60,61]. This prospective registry enrolled 96 consecutive patients undergoing PCI with OA-facilitated lesion preparation. Unlike the longer-term follow-up in previous trials, LOAR focused on mid-term outcomes, with primary endpoints including in-hospital and 6-month major adverse cardiac and cerebrovascular events (MACCE). The study reported an in-hospital MACCE rate of 5.2% and a 6-month MACCE rate of 10.4%, with TLR occurring in 1% of patients and vessel perforation occurring in only one patient (Table 3) [61]. These findings support the safety and efficacy of OA in high-risk, real-world populations, although the relatively small sample size and limited follow-up duration underscore the need for further large-scale studies with extended observation periods.

#### 2.1.6. DIRO Study

The DIRO (direct comparison of RA vs. OA for calcified lesions guided by OCT) trial was a prospective, randomised trial comparing the efficacy and safety of RA versus OA for treating severely calcified coronary lesions [64]. The study enrolled 100 patients, 50 patients in each group, with de novo calcified lesions, defined by an arc >180° on OCT (90% of patients in the RA group, 88% in the OA group) or angiographically moderate to severe calcifications if the OCT catheter could not cross the lesion before any intervention (10% in RA group, 12% in OA group) [64].

The primary findings of the DIRO study indicated that stent expansion assessed by distal reference on OCT was significantly greater in the RA group compared to the OA group (99.5% vs. 90.6%; *p* = 0.02). Additionally, the maximum atherectomy area, based on OCT, was significantly larger in the RA group than in the OA group (1.34 mm^2^ vs. 0.83 mm^2^; *p* = 0.004). Despite these differences in tissue modification and stent expansion, the procedural outcomes and clinical events at 8 months did not differ significantly between the two groups. Both groups demonstrated sufficient vascular healing as assessed by OCT at 8 months post-procedure [64]. Interestingly, despite this being a randomised study, there were significant differences between the RA and OA groups with the RA having a higher frequency of LAD lesions and the OA group having a higher LCx/RCA frequency (RA target vessel: LAD 80%, LCx 8%, RCA 12%; OA target vessel: LAD 58%, LCx 20%, RCA 22%; *p* = 0.06). This could potentially have impacted upon their findings because the LAD tends to have a straighter course than the LCx which makes achieving lumen gain easier and also allows larger RA burrs to be utilised.

The DIRO study’s findings suggest that while RA may offer enhanced tissue modification and greater stent expansion, OA delivers comparable clinical efficacy, as seen in the COAST, ORBIT II, and LOAR studies. This reinforces the importance of individualised lesion assessment and procedural planning when selecting an atherectomy strategy. Both RA and OA appear to be viable options for managing severely calcified coronary lesions, but individual patient factors and lesion characteristics alongside operator experience should guide the choice of an atherectomy device. Notably, the DIRO study did not assess calcium fracture, a potential mechanistic advantage of OA. Additionally, the OAS is less frequently associated with slow-flow phenomena than RA, which may be particularly relevant in patients with severe left ventricular dysfunction or those with long, tortuous, or angulated lesions. Although the study showed a trend toward improved or similar clinical outcomes at 8 months with OA compared to RA—TLR occurred in 4% vs. 6%, TVR in 4% vs. 10%, and major bleeding in 6% vs. 14%—these differences did not reach statistical significance, likely due to the study being underpowered [64]. Lastly, the authors noted that heterogeneity in lesion location distribution may have introduced bias [64].

#### 2.1.7. OAS UK Single-Centre Retrospective Study

A retrospective study by Helal et al. analysed outcomes for 53 patients who underwent OA between 1 January and 31 December 2024, in a high-volume primary PCI centre in the United Kingdom [63]. The study population included patients who were found to have a severely calcified stenosis in a native coronary artery on angiography, IVUS (employed in 18.9%) or OCT (26.4%) that was treated with OA [63].

The findings demonstrated a high procedural success rate of 98.1%. The 30-day MACE rate was 5.7%, with one patient experiencing an in-hospital procedural-related MI and two patients having major bleeding events during follow-up, unrelated to the OAS. Notably, no-flow/slow-flow phenomena were observed in 13.2% of patients, and coronary dissection occurred in 13.2% of cases, all of which were successfully managed with stent implantation (Table 3) [63].

The authors concluded that OA is a safe and effective tool for calcium modification in an all-comers population, supporting its utility in real-world clinical practice. These findings align with previous studies, including the ORBIT II trial, where the procedural success rate was 88.9% [43,63]. However, the study is inherently limited by its retrospective, single-centre design, which introduces potential selection bias and limits the generalisability of the results. The lack of a comparator arm precludes direct comparison against alternative calcium modification techniques, such as rotational atherectomy. Although the OAS was the primary technique employed, adjunctive methods, including RA, IVL and balloon dilatation, were required in 15.1% of cases [63]. This reliance on additional techniques underscores the complexity and heterogeneity of calcified coronary lesions, and reinforces the need for an individualised, lesion-specific interventional approach.

#### 2.1.8. ECLIPSE Trial

The recently presented ECLIPSE (Evaluation of Treatment Strategies for Severe Calcific Coronary Arteries: Orbital Atherectomy Versus Conventional Angioplasty Technique Prior to Implantation of Drug-Eluting Stents) trial was a multicentre, open-label, randomised control trial comparing orbital atherectomy with balloon angioplasty before drug-eluting stent implantation in patients with severely calcified coronary lesions that the operator felt could either be managed with the OAS or standard balloon preparation [62,66]. The aim was to determine whether the OAS could improve outcomes by reducing target vessel failure and increasing minimal stent area. The trial enrolled 2005 patients with 2492 lesions, randomly assigned to either orbital atherectomy (1008 patients) or balloon angioplasty (997 patients). The primary endpoints were target vessel failure (TVF) at 1 year and post-procedural minimal stent area (MSA) at the site of maximal calcification, assessed by OCT in a subset of patients [62].

The findings indicated that TVF at 1 year occurred in 11.5% of patients in the orbital atherectomy group compared to 10.0% in the balloon angioplasty group, with no statistically significant difference (HR 1.16, 95% CI 0.87 to 1.54). Additionally, the mean MSA was not significantly different between the two groups (7.67 mm^2^ for orbital atherectomy vs. 7.42 mm^2^ for balloon angioplasty, *p* = 0.078). Cardiac deaths within 1 year occurred in 39 of 1008 patients in the OA group, and in 26 of 997 in the balloon angioplasty group (*p* = 0.012). OA procedures had longer duration (median 68.0 vs. 52.0 min; *p* < 0.0001), used more contrast volume (median 165.0 vs. 150.0 mL; *p* < 0.0001), and were more likely to have a period of slow flow than balloon angioplasty. There was a numerical, but not statistically significant increase, in perforation frequency with the OAS (18 vs. 10, *p* = 0.14) (Table 3). There was a higher risk of 30 day cardiac death in the OAS cohort (8 vs. 0; *p* = 0.005) but the study was not powered at this timeframe and this signal was lost at one year [62].

One additional important and consistent finding from ECLIPSE was that image-guided PCI was far superior to angiographic guidance. Cases (either OA or balloon dilatation) guided by OCT had vastly improved one year outcomes, thus supporting the essential role of intracoronary imaging, in this case OCT, to guide calcium modification [62].

The authors concluded that routine use of orbital atherectomy before drug-eluting stent implantation did not significantly improve outcomes compared to balloon angioplasty in patients with severely calcified coronary lesions. The study supported a balloon-first approach for most calcified lesions that can be crossed and dilated before stent implantation, guided by intravascular imaging [62]. However, some limitations of the trial warrant consideration. Firstly, the inclusion criteria mandated clinical equipoise between balloon angioplasty and orbital atherectomy, effectively excluding highly complex lesions, including those that were balloon-uncrossable or undilatable. Patients with lesions not deemed eligible for both treatment strategies were excluded, a proportion which was unfortunately not recorded, potentially biasing the results towards those who could be treated effectively with either method. This is further exemplified by the study’s high performance of both treatment groups, achieving far superior MSA results than the study was powered for. The OAS group achieved a median MSA of 7.4 mm^2^ on 5.5 mm^2^ assumed, and similarly balloon angioplasty achieved 7.0 mm^2^ on 4.5 mm^2^ assumed—even the lower quartiles (6.0 mm^2^ and 5.8 mm^2^ respectively) outperforming the assumed medians—suggesting that indeed balloon angioplasty was a viable treatment for the entire cohort, and the statistical insignificance of the OAS presupposed [62]. The significantly better than expected MSA results suggest that the degree of calcification was not as severe and highlight the limitation of utilising angiography to define severe calcification. In current UK clinical practice, the OAS is predominantly employed in cases of severe, complex calcification, particularly lesions that are balloon-uncrossable or undilatable, rather than in the more moderate disease profiles represented in the ECLIPSE cohort. As such, the applicability of ECLIPSE findings to real-world OA use may be limited.

The open-label design of the trial introduces potential for both performance and detection bias, given that treatment allocation was not blinded to operators or patients. Intracoronary imaging guidance was also performed at operator discretion, i.e., non-randomised, which may have affected PCI outcomes. Trial enrolment occurred over 6 years, during which variability in treatment practices and technologies is inevitable. Changes in clinical guidelines and standard care practices over time could have led to variations in patient selection, treatment approaches, and post-procedural care, introducing heterogeneity into the population. In addition, there were a high number of centres in this study enrolling a small number of patients (50 centres enrolling ≤ 10 patients), which raises questions about the volume of OAS cases and the OAS experience in these centres. These factors should be considered when interpreting the results of the ECLIPSE study, as they may limit the generalisability of the findings to broader clinical practice, particularly in patients with longer and more complex calcified lesions. Despite the potential limitations of ECLIPSE, the overriding message is clear that when considering a strategy for managing CAC, particularly when it is relatively less complex, an individualised approach based on patient and lesion characteristics is essential to obtain the best outcomes for each patient.

## 3. Role of Intracoronary Imaging

Intracoronary imaging (ICI) has emerged as an essential tool in optimising procedural outcomes and reducing MACE, particularly in the context of heavily calcified coronary artery disease requiring lesion modification with atherectomies. Effective lesion preparation necessitates the accurate detection and characterisation of CAC, guiding both the need for atherectomy and the choice of adjunctive devices. Utilisation of ICI allows for accurate lesion assessment and measurement, facilitating cases where calcification is not readily apparent on fluoroscopy [36,67,68,69,70].

Studies have demonstrated that intracoronary imaging-guided PCI leads to improved stent expansion and a reduction in MACE, particularly in more complex lesion subsets, and this is now reflected in international guideline recommendations [71,72,73,74,75,76,77,78,79,80,81,82,83]. Both the 2024 European Society of Cardiology (ESC) guidelines and the 2025 American Heart Association (AHA) guidelines advocate for intracoronary imaging, either IVUS or OCT, for procedural guidance during PCI in patients with complex coronary lesions [77,78]. ICI is indicated in addition to angiography for patients with moderate to severe coronary calcification, complex lesions (e.g., left main, bifurcation, long lesions, chronic total occlusions), or when angiography alone is insufficient for PCI guidance [47,68,69,74]. There are various criteria for assessing the significance of calcification on ICI which vary depending on the analysis and also on the mode of ICI (OCT or IVUS). Until recently, the Fujino criteria have been utilised to assess the degree of calcification on OCT. The score allocates two points if the maximum calcium angle is >180°, 1 point if the maximum calcium thickness is >0.5 mm and one point if the calcium length is >5 mm. In their analysis those lesions with a score of 0–3 had excellent stent expansion whereas those with a score of 4 were much more likely to have poor stent expansion (96% vs. 78% *p* < 0.01). More recently the rule of 3’s has been suggested following a retrospective analysis of 250 de novo calcified lesions. One point is allocated for each of: calcium arc of 360°, calcium thickness >3 mm and calcium length > 3 mm with calcium arc of >270°. In a comparative analysis the rule of 3’s was shown to have significantly greater discrimination than the Fujino score (area under the receiver operator curve 0.88–0.90 vs. 0.54, respectively) [44,47,84].

In terms of IVUS a calcium arc > 270°, length ≥ 5 mm, or presence of 360° calcium, especially in vessels < 3.5 mm, indicate need for further modification. Post-modification, visible calcium fractures or cracks are markers of effective preparation [47].

Eruptive and non-eruptive calcified nodules underscore the importance of selecting the right imaging modality. Eruptive nodules are characterised by small calcium fragments that protrude and disrupt the overlying fibrous cap, often accompanied by thrombus [47,85]. Non-eruptive nodules, in contrast, have a smooth, intact fibrous cap with no thrombus [47,85]. These distinct morphologies impact procedural planning. OCT excels in identifying eruptive calcified nodules due to its high spatial resolution, enabling detailed visualisation of cap disruption, calcium protrusion, and thrombus [47,85]. This is vital because eruptive nodules are linked to higher rates of stent edge dissection, incomplete stent apposition, and long-term target lesion failure, despite achieving better acute stent expansion compared to non-eruptive nodules [85].

The further use of ICI post-atherectomy ensures that the calcium has been adequately modified, facilitating optimal stent expansion and reducing the risk of stent-related complications (Figure 1). ICI allows for the sensitive detection of dissections, which can be promptly treated to prevent adverse outcomes [86,87]. There are increasing data to suggest that the increased resolution of OCT may have particular advantages in managing cases with calcification [62,88,89,90]. Overall, intracoronary imaging enables accurate lesion characterisation, informed procedural decision-making, and thorough post-PCI evaluation, rendering it an indispensable tool in delivering personalised PCI strategies in patients with complex coronary disease, including severe calcification.

## 4. Future Perspectives

The interventional cardiology community increasingly recognises the challenges in treating patients with CAC, who often experience poorer short- and long-term outcomes. Severe CAC can hinder stent delivery and expansion, leading to suboptimal procedural outcomes and increased risk of adverse events. Techniques such as RA, OA, ELCA and IVL are employed to modify calcified plaques and facilitate optimal stent implantation.

Notably, the potential synergistic effect of combining different calcium modification tools over device monotherapy, such as RA and IVL, has also been explored in various analyses [91,92,93,94,95,96,97,98,99]. For instance, the Rota-Shock registry study by Sardella et al. evaluated the efficacy and safety of combining RA and IVL for the treatment of severe coronary calcification. In this multicentre study of 160 patients, the primary efficacy endpoint of residual stenosis < 30% was achieved in 96.9% of patients, and the primary safety endpoint was met in 90.6%, with low rates of serious complications. The study thus supports the use of IVL as an elective or bail-out strategy after RA, demonstrating high procedural success and safety in managing severe coronary artery calcification [100]. Further additional studies have shown the potential benefits of a dual calcium modification strategy with IVL [89,97,98,101,102,103]. Collectively, their findings highlight the potential benefits of multimodality calcium modification in PCI, especially in complex and heavily calcified lesions. While ongoing research continues to compare and validate these approaches, evidence on the use of OA in combination strategies remains limited. Orbitaltripsy, a combination of OA and IVL, is an emerging strategy which leverages the complementary mechanisms of OA for initial debulking and luminal gain, followed by IVL for circumferential and transmural calcium fracture, particularly in lesions resistant to single-modality treatment [104,105]. It is potentially useful in cases of extreme calcification, where single-device therapies may be insufficient for optimal stent deployment. Early case reports and small series suggest feasibility, safety, and successful stent expansion, even in complex cases or patients with chronic kidney disease [104,106]. However, further comparative studies and long-term outcomes are needed, and the approach should be tailored to lesion characteristics and procedural goals.

Of the various calcium modification tools available, the OAS in particular has shown promise in enhancing stent delivery and expansion in severely calcified vessels [87,107,108]. However, it has not been definitively proven to improve long-term clinical outcomes compared to other strategies [62,109]. Lesions with both thrombus and extensive calcification in ACS pose additional challenges for atherectomy use. While both OA and RA can aid stent delivery and expansion in calcified lesions, thrombus presence is a relative contraindication due to the increased risk of distal embolisation and no-reflow, especially in ACS with high thrombus burden and vessel fragility [9,16]. This subset of lesions, along with vessel tortuosity, requires careful assessment and individualised planning to minimise complications [9,16].

Beyond the currently available devices, there are further technologies that are in development or in early use for the management of CAC, including Lithix, Javelin and new lithotripsy devices. The Lithix balloon utilises the physics principle of Hertz contact stress using small metal hemispheres within the balloon-based system. Early data from PINNACLE 1 demonstrate the Lithix balloon’s ability to result in multiple deep fractures with optimal stent expansion (103.4% expansion at maximal calcium site) [110]. One of the limitations with the current IVL balloon is that the balloon has a large crossing profile, meaning that it is often not possible to deliver this device to tight of tortuous calcified lesions. The Javelin device is a forward-facing lithotripsy device mounted onto a microcatheter that delivers pulses antegrade to allow modification of calcium immediately in front of the device to enhance the ability to cross the lesion. One of the particular advantages of this device is that it can be advanced over a standard workhorse wire which is favourable to both OA and RA. It is currently being investigated in the FORWARD CAD IDE study [111]. In addition, there are several other lithotripsy balloons that are being developed which may alter the use of this device in future.

Given the limited number of large-scale randomised comparative trials and evolving nature of calcium modification techniques, further research and registries are needed to optimise procedural techniques. An orbital atherectomy registry would provide valuable real-world data on the safety, efficacy, and long-term outcomes of OA, aiding in identifying patient populations that would benefit most from this intervention. Additionally, it would facilitate the development of best practice guidelines and inform clinical decision-making, ultimately improving patient outcomes in the management of calcified coronary lesions.

## 5. Conclusions

Coronary artery calcification remains a common, significant challenge in contemporary PCI, with implications for both immediate procedural success and long-term patient outcomes. Orbital atherectomy, through its dual mechanism of action, offers a promising strategy for the modification of heavily calcified lesions. Ultimately, the choice of calcium modification technique and the use of intracoronary imaging should be individualised, guided by specific patient and lesion characteristics to optimise procedural and clinical outcomes.

## Figures and Tables

**Table 1:** Comparison of Coronary Artery Calcium Modification Devices.

Parameter	Rotational Atherectomy (RA)	Excimer Laser Coronary Atherectomy (ELCA)	Intravascular Lithotripsy (IVL)	Orbital Atherectomy (OA)
Device	Rotablator Rotational Atherectomy System	Spectranetics CVX-300 Excimer Laser System	Shockwave IVL System	Diamondback 360 Coronary Orbital Atherectomy System
Mechanism of Action	Rotational Diamond-tipped burr spins concentrically on the wire Differential cutting ablates calcified plaque	Laser Pulsed ultraviolet laser (308 nm) photoablation, results in vaporisation of plaque Photochemical, photothermal, photomechanical (microbubble formation)	Shockwave Pulsed sonic/acoustic shockwaves fracture calcified plaque intramurally	Orbital Eccentrically mounted diamond-coated crown uses centrifugal force to orbit Differential sanding ablates calcified plaque
Burr/Crown Size	1.25–2.50 mm (Burr)	Catheters with various tip sizes, available with concentric and eccentric tip designs	2.50–4.00 mm (Balloon)	1.25 mm (Crown)
Guidewire	0.009”/0.014” tip RotaWire Guide Wires	0.014” Guidewire	0.014” Guidewire	0.012”/0.014” tip ViperWire Advance Coronary Guide Wire
Ablation Speed	Variable	Adjustable laser energy settings	N/A	80,000 and 120,000 rpm
Ability to ablate forward and backward	No, front-cutting, mono-directional	No, forward emission of laser energy for vaporisation	Circumferential	Yes, bi-directional
Continuous blood flow during ablation	No	Yes	Yes	Yes
Particle Size	5–10 µm	<10 µm	Not applicable	2 µm
Power Source	Pneumatic system	Laser generator: Spectranetics CVX-300	Electrical generator	Electronic system

**Table 2:** Summary of Orbital Atherectomy Studies.

Study	Study Design	Year	Population	Study Duration	Entry Criteria	Primary Endpoint (s)	Key Findings
ORBIT I	Prospective, single-arm, multicentre	2013	50	6 months	De novo calcified coronary lesions determined by angiography or IVUS	Device performance, procedural success, MACE	Device success 98%, procedural success 94%, in-hospital MACE 4%, 6-month MACE 8%
ORBIT II	Prospective, single-arm, multicentre	2014–2016	443	2 years	Severely calcified coronary lesions, determined by angiography only 92%, or based on IVUS 8%	30-day MACE, 1-year MACE, 2-year MACE, procedural success	30-day MACE 10.4%, 1-year MACE 16.4%, 2-year MACE 19.4%, procedural success 88.9%
Lee et al. Real-world Multicentre Registry	Retrospective, multicentre	2016	458	30 days	Severe CAC, enrolled based on the presence of radio-opacities on angiography 100%	30-day MACCE	30-day MACCE 1.7%, all-cause mortality 1.3%, MI 1.1%, TVR 0%
COAST	Prospective, multicentre, single-arm	2020	100	1 year	Severely calcified coronary lesions, determined by angiography only 65%, by IVUS 21%, or determined by OCT 14%	Procedural success, 30-day MACE	Procedural success 85%, 30-day MACE 15%
LOAR	Prospective, single-arm	2023	96	6 months	Severe calcification, determined by angiography or IVUS	In-hospital MACCE, 6-month MACCE	In-hospital MACCE 5.2%, 6-month MACCE 10.4%
DIRO	Prospective, randomised	2023	100	8 months	De novo calcified lesions, assessed by OCT (90% in RA group, 88% in OA group) or angiographically (10% in RA group, 12% in OA group)	Stent expansion, procedural outcomes	RA group had greater stent expansion (99.5% vs. 90.6%)
Helal et al. UK Single-centre Study	Retrospective, single-centre	2025	53	1 year	Severely calcified stenosis in a native coronary artery on angiography 54.7%, IVUS 18.9% or OCT 26.4%	Procedural success, 30-day MACE	Procedural success 98.1%, 30-day MACE 5.7%
ECLIPSE	Prospective, randomised, multicentre	2025	2005	2 years	Severely calcified coronary lesions, confirmed angiographically (97.1% of lesions in OA group, 97.0% in balloon angioplasty group), or by OCT	Target vessel failure, in-stent minimal cross-sectional area	No significant difference in 1-year target vessel failure or minimal stent area between OA and balloon angioplasty groups

IVUS: intravascular ultrasound; OCT: optical coherence tomography; MI: myocardial infarction; MACE: major adverse cardiac event; MACCE: major adverse cardiac and cerebrovascular events; OA: orbital atherectomy; RA: rotational atherectomy; TLR: target lesion revascularisation; TVR: target vessel revascularisation.

**Table 3:** Orbital Atherectomy Safety Outcomes.

Study	Year	Patients (N)	Dissection (%)	Perforation (%)	Slow Flow/No Reflow (%)	Abrupt Closure (%)	In-Hospital MACE (%)	30-Day MACE (%)	30-Day TVR/TLR (%)
ORBIT I	2013	50	12 ^a^	2.0	0	0	4	6	2.0
ORBIT II	2014–2016	443	3.4 ^b^	1.8	0.9	1.8	9.8	10.4	1.4
Lee et al. Real-world Multicentre Registry	2016	458	0.9	0.7	0.7	NS	NS	1.7 ^c^	0.0
COAST	2020	100	2.0	2.0	2.0	3.0	14	15	1.0
LOAR	2023	96	NS	1.0	2.0	1.0	5.2 ^c^	NS	NS
DIRO	2023	100	2.0 in OA	0.0 in RA, 2.0 in OA	6.0 in RA, 4.0 in OA	NS	NS	NS	0.0 in both groups
Helal et al. UK Single-centre Study	2025	53	13.2	0.0	13.2	NS	1.9	5.7	0.0
ECLIPSE	2025	2005	6.9 in OA ^b^, 6.3 in BA ^b^	1.8 in OA, 1.0 in BA	1.7 in OA, 0.5 in BA	0.6 in OA, 0.2 in BA	NS	NS	1.5 in OA, 1.2 in BA

MACE: major adverse cardiac events; TVR: target vessel revascularisation; TLR: target lesion revascularisation; NS: data not specified; OA: orbital atherectomy group; RA: rotational atherectomy group; BA: balloon angioplasty group. ^a^ Dissection types A-C; ^b^ Dissection types C-F, ^c^ MACCE: major adverse cardiac and cerebrovascular events.

## Data Availability

Not applicable.
